# Epidemiological Characteristics and Clinical Outcomes of Coronavirus Disease Patients in Northwest China: High-Volume Research From Low Population Density Regions

**DOI:** 10.3389/fmed.2020.564250

**Published:** 2020-10-30

**Authors:** Jianfei Zhu, Qingqing Zhang, Chenghui Jia, Wuping Wang, Jiakuan Chen, Yanmin Xia, Wenchen Wang, Xuejiao Wang, Miaomiao Wen, Hongtao Wang, Zhipei Zhang, Shuonan Xu, Jinbo Zhao, Tao Jiang

**Affiliations:** ^1^Department of Thoracic Surgery, Tangdu Hospital, Air Force Military Medical University (Fourth Military Medical University), Xi'an, China; ^2^Department of Thoracic Surgery, Shaanxi Provincial People's Hospital, Xi'an, China; ^3^Department of Pulmonary and Critical Care Medicine, The First Affiliated Hospital of Xi'an Medical University, Xi'an, China; ^4^Department of Cardiothoracic Surgery, The First Affiliated Hospital of Xi'an Medical University, Xi'an, China

**Keywords:** COVID-19, low density population, public health intervention, epidemiological characteristics, outcomes

## Abstract

**Background:** Few studies have reported the transmission characteristics of coronavirus disease (COVID-19) in low-density populations. This study has therefore analyzed the epidemiological characteristics and clinical outcomes of COVID-19 patients in Northwestern China, an area with low population density.

**Methods:** From January 21 to March 11, 2020, data from patients diagnosed with novel coronavirus pneumonia (NCP) in areas of Northwestern China with lower population densities were retrospectively analyzed. Certain variables were categorized as numbers and percentages, with the ratio between resident patients (no history of going out during the epidemic) and imported patients representing the contagiousness of severe acute respiratory syndrome coronavirus 2 (SARS-CoV-2) responsible for COVID-19. Hospitalization time was also calculated.

**Results:** A total of 617 COVID-19 patients were reported in Northwestern China, and the morbidity and mortality rates of 0.000005 and 0.011, respectively. Further analysis showed that the morbidity was inversely proportional to population density and distance from Wuhan City. This study enrolled 473 confirmed cases; among these patients, there were 248 residents and 225 imported cases with a ratio of 1:1. The youngest and oldest patients were 1 and 94 years of age, respectively, with a median age of 42 years. Fifteen (3.2%) patients were children or infants. Two patients were pregnant, and one patient gave birth to a healthy baby with negative results during her disease course. About 17.3% of patients (82 cases) were healthy carriers without any symptoms during their disease course. One male patient (0.2%) had recurrence of a positive test result 4 days after discharge. The median hospitalization time was 16.0 days, ranging from 2.0 to 43.0 days. Further analysis showed that age (*P* = 0.03) and severity status (*P* < 0.001) were significantly correlated with hospitalization time.

**Conclusions:** The morbidity and mortality rates of COVID-19 patients in the regions with a low population density were lower than those of the national average in China. All populations were susceptible to infection by SARS-CoV-2. Asymptomatic patients with positive results should be taken seriously, and the hospitalization time of patients is associated with their age and severity status.

## Introduction

Coronavirus disease (COVID-19) (1), also called novel coronavirus pneumonia (NCP) by Chinese officials, emerged in Wuhan City in late December 2019 and remains an ongoing outbreak that has spread globally (2). After critical management of COVID-19 by restricting transmission and treating patients, the number of infected people from China has declined but has sharply increased outside China, especially in Middle Asia, Europe, and the United States (3). On March 3, 2020, the World Health Organization (WHO) reported that more than 132,000 cases had been diagnosed from 123 countries and regions, with more COVID-19 cases reported in Europe every day than were reported in China at the height of its pandemic (4). In addition, recent studies have shown that the infection rate of COVID-19 has been higher than that of Severe Acute Respiratory Syndrome (SARS) (5). To identify the infection source, transmission route, and susceptible population, researchers have focused on investigating the epidemiology of COVID-19 in densely populated areas of China (6, 7); however, only few have reported on regions with lower population densities. Northwestern China is adjacent to Middle Asia and far from the outbreak area; moreover, it has a low population density, inconvenient transport system, and insufficient medical facilities. The potential differences in this epidemic disease in other countries and regions are not clear. We consequently performed this study on the clinical features of COVID-19 cases in these regions compared to epidemiology and clinical prognosis of NCP patients from Northwestern China to contribute to NCP prevention and control and to help other countries similar to the geography and demographic distribution of Northwestern China.

## Materials and Methods

### Study Design

From January 21 to March 11, 2020 (January 24 was the Chinese New Year), all patients diagnosed with NCP from seven provinces or autonomous regions in Northwestern China with a low population density were retrospectively analyzed. The diagnostic criteria were based on the seventh edition of the National New Coronavirus Pneumonia Diagnosis and Treatment Program (8) developed by the National Health Commission of the Peoples' Republic of China. The patient inclusion criteria were the following: (1) positive severe acute respiratory syndrome coronavirus 2 (SARS-CoV-2) result by reverse transcription-polymerase chain reaction (RT-PCR); (2) receiving treatment at a designated hospital; (3) definite outcome (discharge or death); and (4) adequate clinical information and available follow-up data. This study was approved by the ethics committee of Tangdu Hospital. Written informed consent was waived due to the nature of open-access data. The last follow-up was on March 11, 2020.

### Setting and Population Density

Northwestern China is adjacent to Middle Asia, far from the outbreak epicenter. This region has a low population density, inconvenient transport system, and insufficient medical facilities. Northwest China consists of four autonomous regions (Tibet Autonomous Region, Xinjiang Uygur Autonomous Region, Ningxia Hui Autonomous Region, and Inner Mongolia Autonomous Region) and three provinces (Shaanxi Province, Qinghai Province, and Gansu Province) (Table 1). The seven provinces or autonomous regions of Northwestern China cover ~57.5% of China's total territory, with an area of 5,490,400 km^2^; however, its total population from the 2018 census data is only 131,590,000 (9). Hubei Province, located in Central-Eastern China, the COVID-19 outbreak area, has a population density of 318.3 people/km^2^. The population density of Northwestern China is, however, relatively low (23.8 people/km^2^)—lower even than the national average (145.4 people/km^2^). The distances between the capital of the province or the autonomous region and Wuhan City are also shown in Table 1.

**Table 1:** The population and geography features of seven provinces or autonomous regions in Northwestern China.

**Region**	**Distance (km)^a^**	**Population density**	**Confirmed cases^b^**	**Infection density^c^**	**Morbodity^d^**
Shaanxi province	731	187.8	245	0.001	0.000006
Qinghai province	1,597	8.4	18	0.00002	0.000003
Tibet autonomous region	3,568	2.8	1	0.0000008	0.0000003
Xinjiang Uygur autonomous region	3,267	15.0	76	0.00005	0.000003
Ningxia Hui autonomous region	1,468	103.6	75	0.001	0.00001
Gansu province	1,383	58.1	127	0.0003	0.000005
Inner Mongolia autonomous region	1,436	21.5	75	0.00006	0.000003
Hubei province	–	318.3	67,781	0.4	0.001

a Distance: between the capital of province or autonomous region and Wuhan city;

b Confirmed cases: cumulative confirmed cases as at March 11th, 2020;

c *Infection density: Number of confirmed cases/km^2^*;

d *Morbodity: The proportion of confirmed cases in the total population*.

### Data Collection

We obtained data on the exposure history, age, sex, clinical signs and symptoms, diagnosis time, treatment, and outcome from the news reports and press releases reported by the Health Commission of Tibet Autonomous Region, Xinjiang Uygur Autonomous Region, Ningxia Hui Autonomous Region, Inner Mongolia Autonomous Region, Shaanxi Province, Qinghai Province, and Gansu Province. The degree of severity, diagnostic criteria, and discharge criteria refer to the seventh edition of the National New Coronavirus Pneumonia Diagnosis and Treatment Program. All data were collected by Doctors Zhu, Zhang, and Jia, and major disagreements between these three doctors was checked by a fourth reviewer (Doctor Xu). Data are verified with the National Health Commission and the Chinese Center for Disease Control and Prevention.

### Follow-Up

The follow-up of patients after discharge was equally important; all discharged patients should continue to be isolated for medical observation for 14 days. During the isolation period, the body temperature, physical signs, and other conditions should be monitored daily in observation. All patients reviewed the pathogenic test of SARS-CoV-2 at last quarantine day, and nucleic acid testing will be performed at any time if any symptoms appear after discharge.

### Statistical Analysis

Hospitalization time was defined as the time from the final diagnosis to discharge or death. According to severity status, all patients were divided into group for general, severe, and critical (8). Categorical variables are summarized as numbers and percentages. *T*-tests were performed to compare the differences in hospitalization time among groups; when the cases were not normally distributed, Mann–Whitney U or Kruskal–Wallis H-tests were used. A bilateral *P* < 0.05 was considered statistically significant. All analyses were performed using IBM SPSS Statistics for Windows, version 22.0.

## Results

### Epidemiological Characteristics

**Figure 1:**
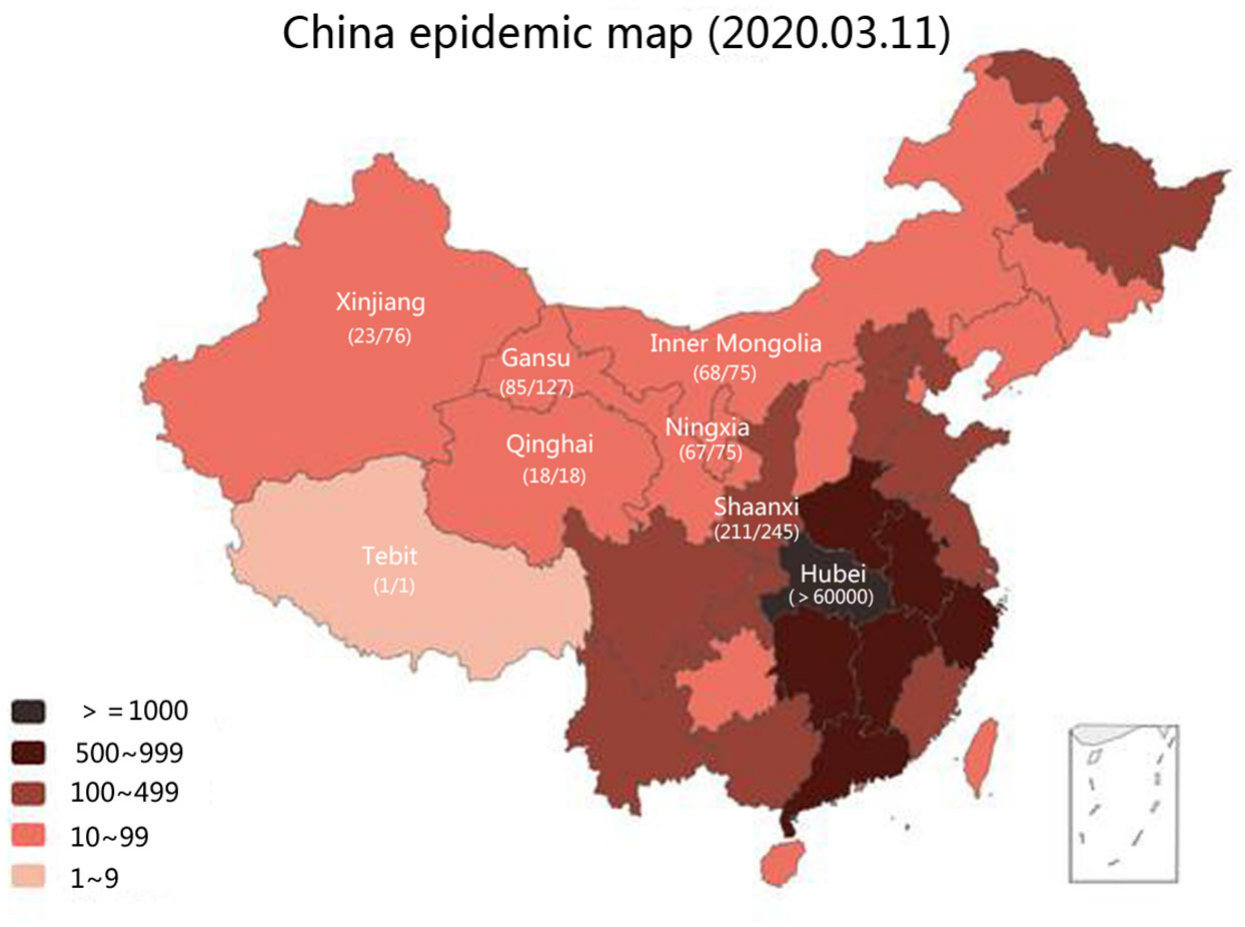
Northwestern China and the epidemic map of China.

**Figure 2:**
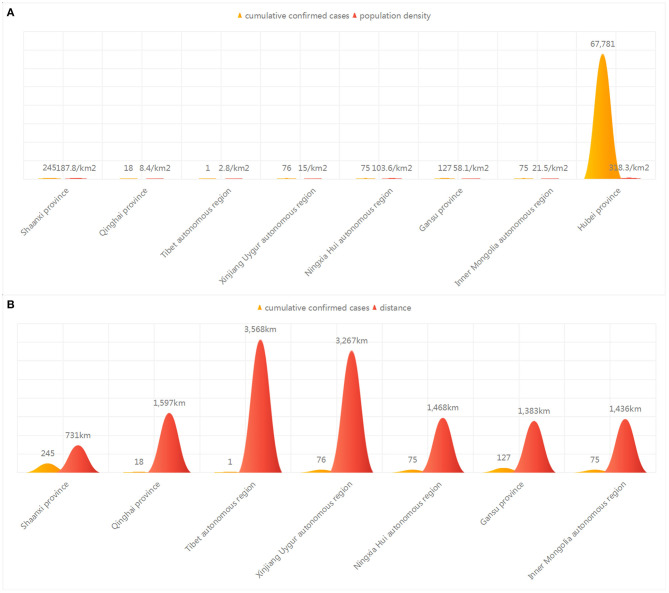
Correlation between geographical factors and COVID-19. **(A)** Distribution of cumulative confirmed cases and population density in Northwestern China. **(B)** Distribution of cumulative confirmed cases and distances between the provincial capital and Wuhan City and Northwestern China.

As of March 11, 2020, 80,793 patients had been diagnosed with COVID-19 in China; of these, 67,781 were in Hubei Province, while 617 were in seven northwestern provinces or autonomous regions, accounting for 0.8%. The morbidity and mortality rates were 0.000005 and 0.011, respectively, in the low population-density region. The epidemic map of China (Figure 1) showed that the cumulative confirmed cases in the seven northwestern provinces or autonomous regions were far less than those in Hubei and other provinces. Figure 2A showed that the number of infected patients increased with population density. The Tibet Autonomous Region, with the lowest population density of China, reported only one imported case, without indigenous secondary cases, with an infection density of only 0.0000008 people/km^2^. There were 245 cases in Shaanxi Province, which is the most densely populated of these seven provinces, with an infection density of only 0.001 people/km^2^. The number of cumulative confirmed cases was associated with the distance between the capitals of the province or autonomous region and Wuhan City (Figure 2B, Table 1).

**Figure 3:**
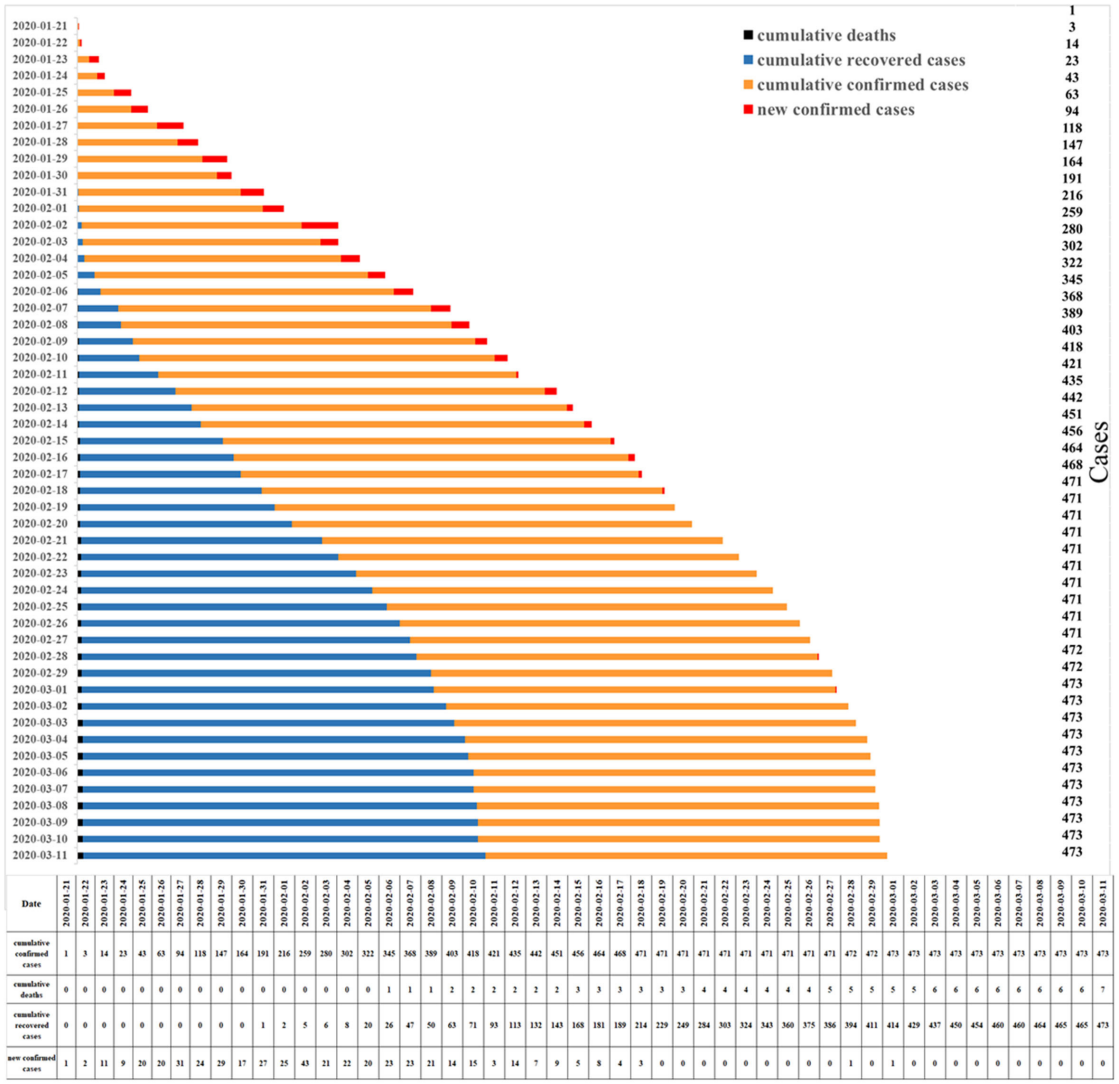
Time distribution of cumulative cases in Northwestern China through March 11th, 2020.

According to the inclusion criteria, a total of 473 patients were finally enrolled in our study for analysis (Table 2). Of the 473 confirmed cases, one was from the Tibet Autonomous Region, 23 from the Xinjiang Uygur Autonomous Region, 67 from the Ningxia Hui Autonomous Region, 68 from Inner Mongolia Autonomous Region, 211 from Shaanxi Province, 18 from Qinghai Province, and 85 from Gansu Province (Figure 1). The first confirmed and discharged patients were reported on January 21 and January 31, 2020, respectively. The peak period for confirmed cases was from January 25 to February 8, 2020 (Figure 3). A total of 194 patients (41.0%) had a history of living or traveling in Hubei Province, 33 patients had an absence of contact history, and two patients had traveled from Iran.

**Table 2:** Clinical characteristics of COVID-19 patients.

**Characteristics**	**N(473)**	**%**
Age, median (range)	42.0 year	1.0–94.0
Gender		
Male	246	52.0%
Female	227	48.0%
Religion		
Shaanxi province	211	44.6%
Qinghai province	18	3.8%
Tibet autonomous region	1	0.2%
Xinjiang Uygur autonomous region	23	4.9%
Ningxia Hui autonomous region	67	14.2%
Gansu province	85	18.0%
Inner Mongolia autonomous region	68	14.4%
Age		
<42	223	47.1%
≥42	250	52.9%
Patient type		
Local cases	248	52.4%
Imported cases	225	47.6%
Exposure history		
Living or traveling in Hubei	194	41.0%
Contact with confirmed patients	244	51.6%
Without contact history	35	7.4%
Symptom		
Fever	255	53.9%
Diarrhea	13	2.7%
Other symptom	123	26.0%
Without symptom	82	17.3%
Severity status		
General	429	90.7%
Severe	26	5.5%
Critical	18	3.8%

### Public Health Interventions and Their Effects

**Figure 4:**
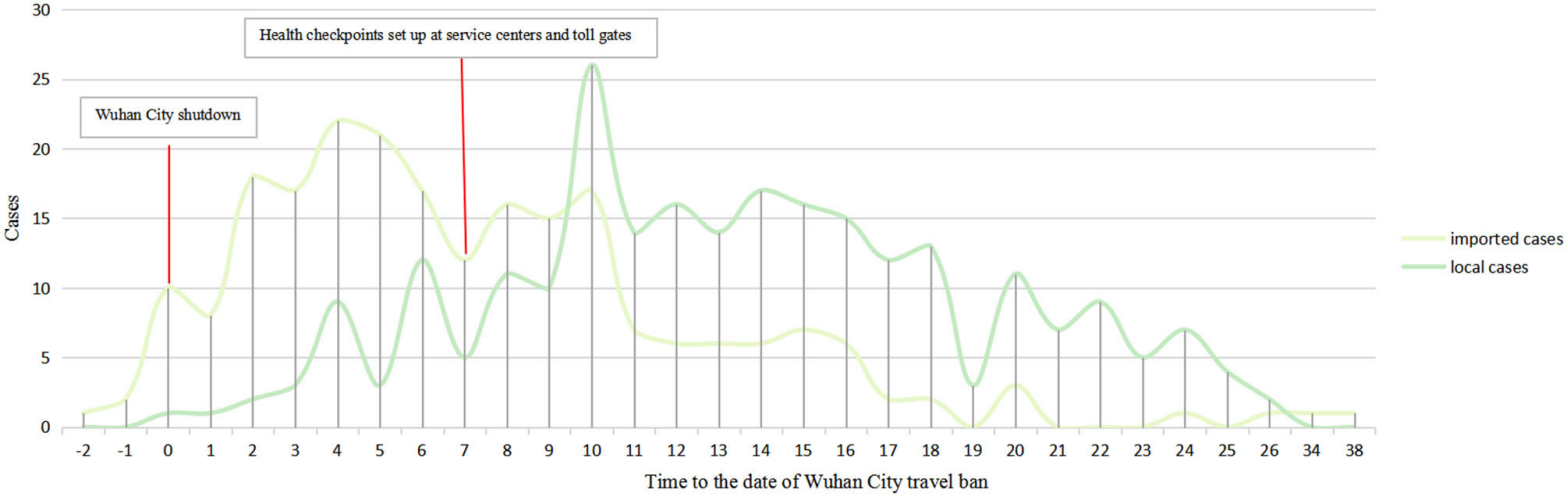
Newly increased COVID-19 cases (local cases vs. imported cases) per day in northwestern China.

From the beginning of the COVID-19 outbreak in Wuhan, the confirmed cases in Northwestern China have gradually been on the increase. On January 21, 2020, the first imported patient emerged, with confirmed local cases increasing with the growth of imported cases within 6 days despite the shutdown of Wuhan on January 23, 2020. Four days after the Wuhan shutdown, the number of new imported patients peaked at 22 cases. Approximately 76.9% of imported cases (173/225) were diagnosed within 10 days of the closure of Wuhan City. To better prevent and control COVID-19 spread, strict countermeasures designed and approved by local authorities were implemented on January 30, 2020, which included raising the public health response level to Class A, setting up health checkpoints in public areas, and locking down the regional border. The daily numbers of imported cases decreased, and more new local cases were confirmed 3 days after the implementation of these control measures. On February 2, 2020, the number of new local patients surpassed imported patients and simultaneously peaked. The next day, the numbers of imported and local patients had significantly decreased and on February 18, 2020 no newly confirmed patients were reported. On February 26, 2020, however, the first imported case from overseas was reported in Northwestern China. The details of the public health events, government anti-virus measures, and related dynamic results are separately displayed in Figure 4.

### Clinical Characteristics

The final analysis included 473 patients (246 men and 227 women). The youngest and oldest patients were 1 and 94 years of age, respectively, with a median age of 42 years. Fifteen patients (3.2%) were <12 years of age. Two patients were pregnant. One of them was full-term. She was infected with SARS-CoV-2 during the delivery period and eventually gave birth to a baby girl. Among all patients, 82 (17.3%) were asymptomatic in their disease course. Fever was the most common symptom (53.9%), ~2.7% of patients presented with diarrhea, and 26.0% of patients with other symptoms. According to severity status, 429 patients (90.7%) were general, 26 patients (5.5%) were severe, and 18 patients (3.8%) were critical. Detailed patient information is listed in Table 2.

### Clinical Outcomes

The current discharge criteria are based on the seventh edition of the COVID-19 diagnosis and treatment guidelines (8). These include the disappearance of clinical symptoms and two consecutive negative nucleic acid tests with an interval time of over 24 h. Of the 473 patients, 466 met the discharge criteria. One male patient (0.2%) had a recurrence of positive test 4 days after discharge.

From March 11, 2020, seven patients died, corresponding to a mortality rate of 1.1% (7/617), which is lower than that of the national average (3.9%) (10); the other patients were all discharged (Figure 3). The median hospitalization time was 16.0 days, ranging from 2.0 to 43.0 days. Further analysis showed that age (*P* = 0.03) and severity status (*P* < 0.001) were significantly correlated with hospitalization time (Table 3). While patients presenting with diarrhea had shorter hospitalization times than those of patients presenting the other symptoms, the difference was not of statistical significance (16.0 vs. 17.0 vs. 14.0 vs. 14.0 days, *P* = 0.091).

**Table 3:** Clinical outcomes of COVID-19 patients.

**Variable**	***N***	**Hospitalization time (days)**	**Percentiles**	***P***
In total	473	43.0	–	
Gender				0.323
Male	246	17.0	(12.0, 21.0)	
Female	227	16.0	(11.0, 20.0)	
Age				0.030
<42	223	16.0	(11.0 ,20.0)	
≥42	250	17.0	(12.0, 21.0)	
Patient type				0.196
Local cases	248	16.0	(11.0, 20.0)	
Imported cases	225	17.0	(12.0, 21.0)	
Exposure history				0.303
Living or traveling in Hubei	194	17.0	(12.0, 21.0)	
Contact with confirmed patients	244	16.0	(11.0, 21.0)	
Without contact history	35	16.0	(11.0, 20.0)	
Symptom				0.091
Fever	255	17.0	(13.0, 21.0)	
Diarrhea	13	14.0	(10.75, 21.5)	
Other symptom	123	16.0	(12.0, 20.0)	
Without symptom	82	14.0	(10.75, 20.0)	
Severity status				<0.001
General	429	16.0	(11.5, 20.0)	
Severe	26	20.5	(14.75, 26.0)	
Critical	18	23.5	(16.25, 32.25)	

Of the seven patients who died, four were male and three were female (Table 4). Only one patient was younger (aged 48 years); the other patients were aged over 70 years (70, 73, 76, 77, 82, and 89 years). According to disease severity two and five of these patients were categorized as severe and critical, respectively. Only two patients died without comorbidity; three patients were diagnosed with hypertension, and two patients were diagnosed with chronic obstructive pulmonary disease (COPD).

**Table 4:** Clinical characteristics of seven patients died from COVID-19.

**No**	**Age**	**Gender**	**Exposure history**	**Symptom**	**Severity status**	**Comorbidity**
1	89.0	Male	Contact with confirmed patients	Cough	Critical	Hypertension, cerebral infarction, gout
2	82.0	Female	Contact with confirmed patients	Cough	Severe	Hypertension, Alzheimer disease
3	77.0	Male	Living or traveling in Hubei	Without symptom	Critical	Hypertension, COPD
4	73.0	female	Contact with confirmed patients	Fever	severe	Hypertension
5	76.0	Female	Without contact history	Without symptom	Critical	Hypertension, Atrial fibrillation, Chronic bronchitis
6	70.0	Male	Without contact history	Without symptom	Critical	Without
7	48.0	Male	Without contact history	Fever, cough and diarrhea	Critical	Without

## Discussion

Three months ago, the COVID-19 outbreak started in Wuhan City (1), the capital of Hubei Province, which is located in the central region of China and is known as “Chinese Chicago”—a developed transportation hub for advanced railway, water carriage, and aviation systems. The population density of Wuhan is 1,249 people/km^2^. The main transmission routes of SARS-CoV-2 include droplets and aerosols (11). Compared to other infectious diseases, such as SARS and Middle East Respiratory Syndrome (MERS), COVID-19 thus has higher infectivity (5). A previous study proved that high population density catalyzes the spread of COVID-19 (12). To our knowledge, the present was the first study to assess the impact of government public health interventions on the spread of COVID-19 in low population density areas. A total of 617 patients were identified in these regions, accounting for 0.8% of the total number of Chinese cases. The prevalence of COVID-19 in Northwest China was 0.000005, which is lower than the national average (0.00006).

The lower morbidity in Northwest China might be attributed to its low population density and geographical distance from the epicenter. A previous study proved that high population densities catalyze the spread of COVID-19 (12), a finding consistent with our results. In our study, compared to high-density areas, low-density areas had lower morbidity. In addition to population density, the regions and autonomous regions in this study are far from the urban center of China. Compared to other regions closer to Hubei, the lower incidence in the seven northwestern provinces farther from Wuhan was related to the geographical distance. Our findings confirmed that Xinjiang and Tibet Autonomous Region, far from Wuhan, had a much lower incidence than in Shaanxi Province, which is relatively close to Wuhan. The traffic conditions and proportion of floating population in Wuhan also implied that distance is a factor affecting the epidemic (13).

Moreover, public health interventions, advocated by the government, also played an important role in preventing and controlling the spread of COVID-19. Tian et al. reported that the Wuhan City shutdown delayed the spread of COVID-19 to other cities for 2.91 days (95% confidence interval [CI]: 2.54–3.29 days) (14). A simulation analysis by Chinese scholars proved that both quarantine and traffic blockage were significant methods to control the outbreak (15). The economic level, the ability of government management and the activities of medical treatment have great impact on the incidence rate and mortality rate of different regions (16, 17). In our study, public health interventions designed and approved by local authorities included setting up health checkpoints in public areas and locking down the regional border. Four days after the implementation of control measures, the number of imported and local cases decreased significantly, and no newly confirmed cases occurred after 19 days, Surprisingly, in the first 3 days after restricting activities, the number of new cases increased, which may be related to the familial spread of COVID-19. Related research confirmed family clustering sin the spread of COVID-19 (18).

In addition, quarantine and medical observation of potentially infected populations are effective methods of epidemic control (19), especially in screening asymptomatic patients. It remains controversial whether asymptomatic patients are contagious. A study from Henan Province of China showed that an asymptomatic carrier transmitted COVID-19 virus to her five family members (20); therefore, the management of asymptomatic patients is critical for preventing outbreaks. We reported that ~17.3% of patients were asymptomatic carriers, all of whom were detected during the period of quarantine or medical observation.

Early research has suggested that infants and young children belong to a disease-exempt population. However, the results of later studies (21, 22) refuted these initial findings. In our study, the youngest confirmed patient was only 1 year of age and 15 infants or children accounted for 3.2% of the total cases. It is worth noting that the confirmed specimen was a stool sample from a 1-year-old child, which indirectly proved the possibility of fecal–oral transmission of COVID-19, especially in children (23, 24).

In China, the current discharge criteria are based on the seventh edition of the COVID-19 diagnosis and treatment guidelines (8). These consist of the disappearance of clinical symptoms two consecutive negative nucleic acid test results at least 24 h apart. There are, however, sporadic case reports of recurrence worldwide (25), and we also observed one patient (0.2%) with recurrence of positive test 4 days after discharge.

The mortality rate of patients diagnosed with COVID-19 in this study was 1.1%, which was significantly lower than those reported in Wuhan City, Europe, and Central Asia (3). A possible reason for the low mortality rate is that, although the medical resources of Northwestern China are relatively insufficient compared to those of the eastern regions of China and European countries, every single patient can receive sufficient medical support, and most of the cases were imported young patients who left Wuhan City for work and had returned home (13).

The limitations of this study cannot be ignored. First, the laboratory results of patients could not be further analyzed due to the data came from news reported by the CDC in the provinces and autonomous regions; Second, although we have compared the incidence and mortality of COVID-19 in low-density areas with the national average, the data in other high-density areas outside the epidemic area were deficient; Finally, further analysis was limited because of the small sample size of this study.

In summary, the results of this study revealed that population density and distance from the epicenter are important factors affecting the prevalence of COVID-19. Public health interventions are useful for COVID-19 prevention and control. Additionally, the management of asymptomatic patients and children is more efficient when attempting to restrict the outbreak. Finally, the hospitalization time of patients was associated with their age and severity status.

## Data Availability Statement

The original contributions presented in the study are included in the article/supplementary materials, further inquiries can be directed to the corresponding author/s.

## Ethics Statement

The study was approved by the review board of Tangdu hospital of Air Force Medical University. Written informed consent for participation was not required for this study in accordance with the national legislation and the institutional requirements.

## Author Contributions

TJ, JZha, and SX participated in study design and study conception. JZhu, QZ, WuW, JC, YX, WeW, XW, MW, HW, and ZZ performed data analysis. JZhu, QZ, CJ, and SX recruited patients. JZhu, QZ, CJ, WuW, and JC drafted the manuscript. All authors provided critical review of the manuscript and approved the final draft for publication.

## Conflict of Interest

The authors declare that the research was conducted in the absence of any commercial or financial relationships that could be construed as a potential conflict of interest.
